# 高致敏单倍体造血干细胞移植患者采用蛋白A免疫吸附联合利妥昔单抗行脱敏治疗的疗效及安全性

**DOI:** 10.3760/cma.j.cn121090-20231125-00277

**Published:** 2024-05

**Authors:** 玲 李, 文娟 朱, 倩 朱, 士源 周, 超 马, 俊 王, 晓慧 胡, 悦 韩, 荧 王, 晓文 唐, 骁 马, 苏宁 陈, 惠英 仇, 璐瑶 陈, 军 何, 德沛 吴, 小津 吴

**Affiliations:** 1 苏州大学附属第一医院血液内科，国家血液系统疾病临床医学研究中心，江苏省血液研究所，苏州大学造血干细胞移植研究所，苏州 215006 Department of Hematology, The First Affiliated Hospital of Soochow University, Jiangsu Institute of Hematology, National Clinical Research Center for Blood Diseases, Institute of Hematopoietic Stem Cell Transplantation, Soochow University, Suzhou 215006, China; 2 苏州弘慈血液病医院，苏州 215128 Hematology Department, Soochow Hopes Hematonosis Hospital, Suzhou 215128, China

**Keywords:** 免疫吸附技术, 造血干细胞移植, 脱敏治疗, 利妥昔单抗, Immunosorbent techniques, Hematopoietic stem cell transplantation, Desensitization, treatment, Rituximab

## Abstract

**目的:**

探究高致敏单倍体造血干细胞移植（haplo-HSCT）患者移植前行蛋白A免疫吸附（PAIA）联合利妥昔单抗（RTX）脱敏治疗的疗效及安全性。

**方法:**

回顾性分析2021年3月至2023年6月苏州大学附属第一医院和苏州弘慈血液病医院收治的高致敏haplo-HSCT患者56例，移植前行PAIA联合RTX脱敏治疗，吸附前后监测HLA抗体种类数量和平均荧光强度（MFI）、体液免疫和吸附过程的不良反应及100 d内的生存情况。

**结果:**

仅含HLA Ⅰ类抗体的患者接受PAIA治疗后中位MFI由7 859（3 209～12 444）降至3 719（0～8 275）（*P*<0.001），HLA Ⅰ+Ⅱ类抗体的中位MFI由5 476（1 977～12 382）降至3 714（0～11 074）（*P*＝0.035），其中抗供者特异性抗体阳性患者的中位MFI由8 779（2 697～18 659）降至4 524（0～15 989）（*P*<0.001）。所有患者HLA-A、B、C、DR、DQ抗体种类数量在PAIA治疗后均下降，差异均有统计学意义（A、B、C、DR：*P*<0.001，DQ：*P*<0.01）。PAIA治疗前后体液免疫监测显示IgG和补体C3数量显著下降（*P*值分别为<0.001和0.002）。44例患者接受了移植后HLA抗体监测，总体MFI和抗体种类数量均下降，但有5例患者出现低MFI的新生抗体，有9例持续高MFI，移植后100 d总生存率是83.8％，100 d内无病生存率为80.2％，100 d内非复发死亡率为16.1％，移植后100 d复发的累积发生率为4.5％。

**结论:**

PAIA联合RTX对haplo-HSCT前高敏患者的脱敏治疗有一定的疗效，安全性良好。

## Abstract (en)

**Objective:**

To investigate the efficacy and safety of protein A immunoadsorption（PAIA）combined with rituximab（RTX）in highly sensitized patients who underwent haplo-hematopoietic stem cell transplantation（haplo-HSCT）.

**Methods:**

The clinical data of 56 highly sensitized patients treated with PAIA and RTX before haplo-HSCT at the First Affiliated Hospital of Soochow University and Soochow Hopes Hematonosis Hospital between March 2021 and June 2023 were retrospectively analyzed. The number of human leukocyte antigen（HLA）antibody types and the mean fluorescence intensity（MFI）, humoral immunity, adverse reactions during adsorption, and survival within 100 days before and after adsorption were measured.

**Results:**

After receiving the PAIA treatment, the median MFI of patients containing only HLA Ⅰ antibodies decreased from 7 859（3 209–12 444）to 3 719（0–8 275）（*P*<0.001）, and the median MFI of HLA Ⅰ+Ⅱ antibodies decreased from 5 476（1 977–12 382）to 3 714（0–11 074）（*P*＝0.035）. The median MFI of patients with positive anti-donor-specific antibodies decreased from 8 779（2 697–18 659）to 4 524（0–15 989）（*P*<0.001）. The number of HLA-A, B, C, DR, and DQ antibodies in all patients decreased after the PAIA treatment, and the differences were statistically significant（A, B, C, DR: *P*<0.001, DQ: *P*<0.01）. The humoral immune monitoring before and after the PAIA treatment showed a significant decrease in the number of IgG and complement C3（*P*<0.001 and *P*＝0.002, respectively）. Forty-four patients underwent HLA antibody monitoring after transplantation, and the overall MFI and number of antibody types decreased. However, five patients developed new antibodies with low MFI, and nine patients continued to have high MFI. The overall survival, disease-free survival, non-recurrent mortality, and cumulative recurrence rates at 100 days post-transplantation were 83.8%, 80.2%, 16.1%, and 4.5%, respectively.

**Conclusion:**

The combination of PAIA and RTX has a certain therapeutic effect and good safety in the desensitization treatment of highly sensitive patients before haplo-HSCT.

近三年来，全国造血干细胞移植数量已经突破了10 000例/年，30％～40％急性白血病患者存在抗HLA抗体，抗供者特异性抗体（DSA）是单倍体造血干细胞移植（haplo-HSCT）植入失败的重要原因，移植前行有效的脱敏治疗非常有必要[1]–[2]。目前主要的脱敏治疗方法为利妥昔单抗（RTX）联合血浆置换，但存在血浆紧缺、血液制品输注导致感染等问题。蛋白A免疫吸附（protein A immunoadsorption，PAIA）是一种通过体外循环方式进行血液成分净化的疗法，患者的血浆流经蛋白A吸附柱，致病免疫球蛋白被特异性吸附，主要清除IgG自身抗体和免疫复合物，最后将净化血浆回输至患者体内，PAIA的特异性通常优于血浆置换。PAIA既往主要用于实体器官移植（如肾移植）、自身免疫性疾病致病抗体的清除，在造血干细胞移植中的应用尚未被广泛报道。苏州大学附属第一医院和苏州弘慈血液病医院尝试在移植前采用PAIA联合RTX治疗高致敏haplo-HSCT患者56例，本研究回顾性分析了PAIA联合RTX的疗效、安全性及对移植结果可能产生的影响。

## 病例与方法

1. 病例：回顾性分析了2021年3月至2023年6月在苏州大学附属第一医院和苏州弘慈血液病医院接受haplo-HSCT的56例高致敏患者，均接受了PAIA治疗，其中男8例，女48例。中位年龄41（24～66）岁。所有患者移植前HLA抗体检测平均荧光强度（MFI）最大值≥8 000，这些抗体均为DSA或与DSA有交叉反应的抗原对应的抗体。收集患者的性别、年龄、疾病、移植前疾病状态、移植相关资料（预处理方案、供受者关系、供受者血型、供受者性别等）、移植结果、移植后并发症及移植后100 d内患者的生存情况。

2. 抗HLA抗体的测定：所有受者均在移植前进行抗HLA抗体混合筛查。采用免疫微珠液相芯片技术检测患者血清中抗HLA抗体的种类及强度。抗体混合筛查阳性患者进一步行HLA抗体特异性检测，包括HLA Ⅰ类（HLA A、B、C）和HLA Ⅱ类（HLA DR、DP、DQ）。抗HLA抗体混合初筛检测中，MFI的临界值设定为500。测定时间点分别为脱敏治疗前（T0）、脱敏治疗后（T1）、移植后（T2）。其中T0为未行治疗时的抗体测定时间，T1是脱敏治疗后最接近移植的时间点，T2是移植后最后一次随访的时间。

3. 脱敏治疗方法：所有受者移植前均接受以PAIA及RTX为主的脱敏治疗方案。预处理的2周内开始PAIA治疗，隔日治疗1次，根据抗体类型和MFI变化，予2～6次PAIA治疗，治疗流程详见图1。采用蛋白A免疫吸附柱（康碧，KCIA08，广州康盛生物科技股份有限公司产品）完成治疗。患者建立体外循环后，予低分子肝素抗凝，体外循环下全血以100～120 ml/min的速度流经血浆分离器，分离出的血浆再以30～40 ml/min的速度流经葡萄球菌蛋白A免疫吸附柱进行吸附。通过预冲-吸附-回浆再冲洗-洗脱-平衡-二次预冲等6个步骤组成吸附循环，每次6～10个循环，每次吸附治疗血浆3 600～6 000 ml[3]–[4]。吸附过程中注意监测患者的生命体征，有无休克、过敏、心律失常等不适，如有不适则终止治疗。移植前10 d开始应用RTX清除体内B细胞，剂量为375 mg/m^2^，具体治疗流程详见图1。

**图1:**
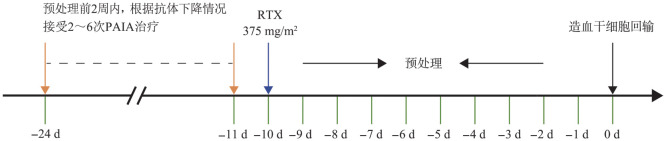
高致敏单倍体造血干细胞移植患者脱敏治疗流程图 **注** PAIA：蛋白A免疫吸附；RTX：利妥昔单抗

4. 移植预处理方案及移植物抗宿主病（GVHD）预防：预处理方案包括改良BuCy方案和FBM方案。改良BuCy方案：白消安（Bu）0.8 mg·kg^−1^·（6 h）^−1^，静脉滴注，−7 d～−5 d；阿糖胞苷（Ara-C）2 g·m^−2^·（12 h）^−1^，静脉滴注，−9 d～−8 d；环磷酰胺（CTX）1.8 g/m^2^，静脉滴注，−4 d～−3 d；加用抗胸腺细胞免疫球蛋白（ATG）预防GVHD，2.5 mg/kg，静脉滴注，−5 d～−2 d。FBM方案：氟达拉滨（Flu）30 mg/kg，静脉滴注，−7 d～−3 d；Ara-C 2 g·m^−2^·（12 h）^−1^，静脉滴注，−9 d～−8 d；Bu 0.8 mg·kg^−1^·（6 h）^−1^，静脉滴注，−6 d～−5 d；美法仑100 mg/kg，静脉滴注，−4 d～−3 d。GVHD预防方案参考文献[5]。

5. 安全性评估和处理：所有接受治疗的患者行心电监护。对于治疗中出现过敏的轻症患者，若降低血流量、抗组胺类药物可缓解，则不需终止治疗；对于重症患者，应立即终止治疗，予适量生理盐水扩充血容量，同时予抗组胺类药物。治疗中出现低血压时，适当补充胶体溶液，缓慢调整血流量。如治疗后患者出现免疫球蛋白低下，吸附后IgG<2.0 g/L时，可视患者具体情况选择补充小剂量丙种球蛋白2.5～5.0 g/次，24 h后再继续治疗。

6. 定义：高致敏：接受异基因造血干细胞移植前患者存在1个及以上位点的抗HLA抗体MFI ≥ 8 000（排除天然抗体），包括受者针对供者特异性位点产生的抗体（DSA）。脱敏治疗无效：脱敏治疗前后预存抗体MFI持续≥8 000。移植后新生抗体：移植前及PAIA治疗后均不存在，在移植后监测中有两次及以上超过检测阳性值。原发性移植失败：缺乏初始供者细胞植入（供受者嵌合度不足95％），在移植后第28天外周血ANC<0.5×10^9^/L，无复发。植入功能不良：移植28 d后出现两系或三系细胞计数未达到植活标准持续2周以上，骨髓检查提示骨髓增生低下，原发病处于缓解状态，细胞为完全供者嵌合，无严重GVHD和复发[6]。中性粒细胞植入标准：ANC>0.5×10^9^/L连续3 d。血小板植入标准：PLT>20×10^9^/L连续7 d且脱离血小板输注。总生存（OS）时间定义为自移植之日起至末次随访或因任何原因死亡的时间。无病生存（DFS）时间定义为自移植之日起至患者复发、死亡、原发性植入失败或末次随访的时间。非复发死亡率（NRM）定义为除复发外的任何原因导致死亡的累积发生率。复发定义为完全缓解（CR）后外周血重新出现白血病细胞或骨髓原始细胞>5％（除外其他原因如巩固化疗后骨髓重建等）或髓外出现白血病细胞浸润。

急性及慢性GVHD的诊断标准参照文献[7]。

7. 随访：随访截止日期为2023年6月30日。通过查阅患者住院病历、门诊随访记录和电话随访获得患者生存资料。中位随访时间为210（4～672）d。

8. 统计学处理：采用Graphpad Prism软件进行统计分析。免疫吸附前后抗体MFI变化采用中位数（范围）表示。采用Kaplan-Meier法进行生存分析。*P*<0.05为差异有统计学意义。

## 结果

1. 一般基线资料：共56例患者纳入研究，男8例，女48例，中位年龄41（24～66）岁。28例诊断为急性髓系白血病，6例诊断为急性淋巴细胞白血病，9例诊断为骨髓增生异常综合征，9例诊断为再生障碍性贫血，4例为其他疾病。诊断为急性白血病的34例患者中，25例患者移植前疾病状态为完全缓解（CR），9例（26.5％）未缓解。42例（75％）患者行清髓性方案预处理，14例（25％）患者行减低强度方案预处理。中位单个核细胞（MNC）输注数量为11.86（4.53～40.26）×10^8^/kg，中位CD34^+^造血干细胞输注数量为5.33（1.22～11.71）×10^6^/kg。

2. 抗体分布情况：56例患者中21例患者仅存在HLA Ⅰ类抗体，中位MFI为5 560（2 430～18 659），4例仅存在HLA Ⅱ类抗体，中位MFI为7 859（3 209～12 444），31例同时存在HLA Ⅰ+Ⅱ两类抗体，中位MFI为5 476（1 977～12 382）。仅HLA Ⅰ类抗体阳性的患者中位抗体种类数量30（7～57），HLA B类抗体占70.0％；HLA Ⅱ类抗体中位抗体种类数量13（9～23），HLA DP类抗体占40.2％；HLA Ⅰ+Ⅱ类抗体中位抗体种类数量37（8～72），HLA A+B+DR类抗体占58.3％。24例患者同时存在DSA，DSA中位MFI 8 779（2 697～8 779），其中HLA Ⅰ类14例，HLA Ⅱ类8例，HLA Ⅰ+Ⅱ类2例。

3. PAIA治疗前后和移植前后抗体MFI和位点数变化：21例仅含HLA Ⅰ类抗体的患者在PAIA治疗后总体中位MFI较吸附前明显下降，中位MFI由治疗前的7 859（3 209～12 444）降至治疗后的3 719（0～8 275）（*P*<0.001）（图2A）。4例仅含HLA Ⅱ类抗体患者PAIA治疗后总体MFI未见明显下降（*P*＝0.250）（图2A）。31例含HLA Ⅰ+Ⅱ类抗体患者PAIA治疗后总体MFI显著下降，中位MFI由5 476（1 977～12 382）降至3 714（0～11 074）（*P*＝0.035）（图2A）。DSA阳性患者PAIA治疗后MFI显著下降，中位MFI由8 779（2 697～18 659）降至4 524（0～15 989）（*P*<0.001）（图2B）。仅含HLA Ⅰ类抗体组和HLA Ⅰ+Ⅱ类抗体组抗体数量在PAIA治疗后均明显下降（HLAⅠ：*P*<0.001，HLA Ⅰ+Ⅱ：*P*<0.001），对不同位点吸附前后抗体种类数量进行比较，A、B、C、DR、DQ等抗体种类数量在吸附后均下降（A、B、C、DR：*P*<0.001，DQ：*P*<0.01）（图3）。

**图2:**
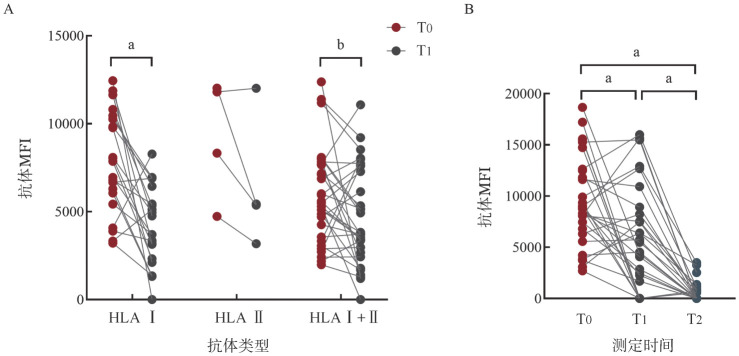
蛋白A免疫吸附（PAIA）治疗前后患者抗体平均荧光强度（MFI）变化 **A** PAIA治疗前（T0）、PAIA治疗后（T1）仅含HLA Ⅰ抗体、HLA Ⅱ抗体、HLA Ⅰ+Ⅱ抗体患者抗体MFI变化；**B** DSA阳性患者PAIA治疗前（T0）、PAIA治疗后（T1）、移植后（T2）抗体MFI变化 **注** ^a^*P*<0.001；^b^*P*<0.05

**图3:**
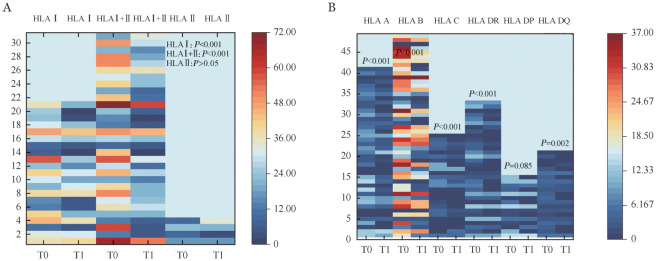
56例患者蛋白A免疫吸附（PAIA）治疗前后抗体种类数量变化 **A** HLA Ⅰ、HLA Ⅱ及HLA Ⅰ+Ⅱ类抗体种类数量变化；**B** HLA A、B、C、DR、DP、DQ抗体种类数量变化 **注** 横坐标表示不同时间点，T0：PAIA治疗前，T1：PAIA治疗后；纵坐标表示患者例号，图例表示位点数目

4. 移植后抗体检测：44例患者移植后接受抗体检测，其中19例患者接受过2次及以上抗体检测，中位检测时间为移植后24（7～180）d，总体抗体中位MFI在移植后明显下降，中位MFI由治疗前的6 364（1 977～12 444）降至移植后的1 639（0～8 606）（*P*<0.001）。9例患者抗体的MFI在移植后仍>8 000，其中3例患者移植后抗体MFI回升，6例患者为抗体持续无效。35例患者移植后抗体MFI及抗体种类数量显著下降，MFI均小于3 000，其中5例出现低丰度（MFI：1 000～3 000，位点数1～2个）的新生抗HLA抗体。24例PAIA治疗前DSA阳性患者的中位MFI在移植后显著下降［8 779（2 697～18 659）对0（0～3 505），*P*<0.001］，移植前的中位MFI与移植后相比差异有统计学意义［4 524（0～15 989）对0（0～3 505），*P*<0.001］（图2B），其中仍有8例患者DSA抗体阳性，中位MFI为931（564～3 505），MFI>2 000的患者有2例。

5. PAIA治疗过程中的不良反应：35例患者接受了PAIA治疗前后免疫球蛋白检测，结果显示免疫球蛋白明显下降，尤其是IgG（*P*<0.001），同时还观察到补体C3下降（*P*＝0.002）（图4），所有患者在吸附过程中均出现轻度血压下降，2例患者严重低血压，所有患者未发生过敏反应，吸附后无肝肾功能损害。所有患者在RTX治疗过程中未见明显不良反应。

**图4:**
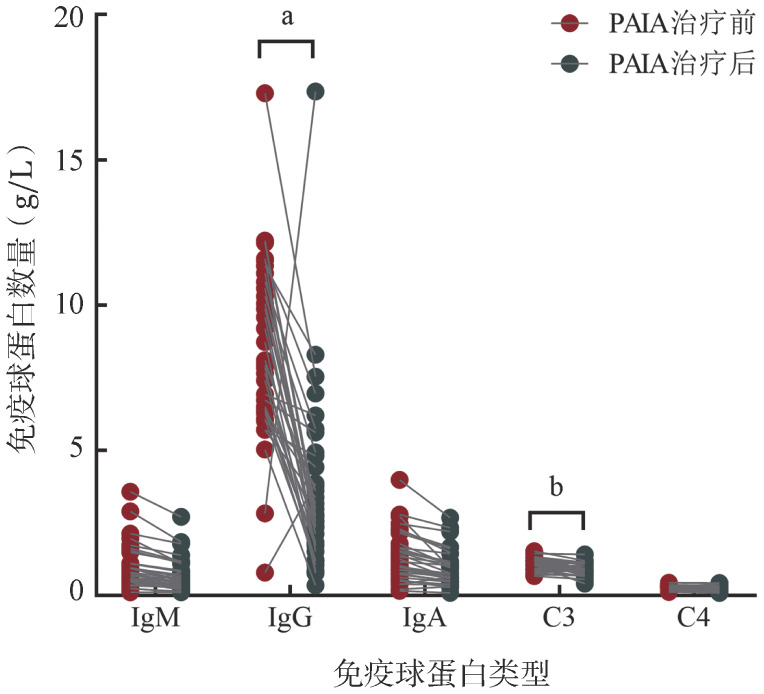
35例患者蛋白A免疫吸附（PAIA）治疗前后免疫球蛋白数量变化 **注** ^a^*P*<0.001；^b^*P*<0.05

6. 移植结果和预后：所有患者中位血小板植入时间为18（4～207）d，中性粒细胞中位植入时间为12（4～41）d。血小板及粒系28 d植入的累积发生率分别为82.9％、94.4％，其中有1例患者发生原发性植入失败，6例患者发生血小板植入功能不良。100 d内Ⅱ～Ⅳ级急性GVHD的累积发生率为13.4％，移植后1年内慢性GVHD累积发生率为22.5％，100 d巨细胞病毒（CMV）感染累积发生率为50.9％，移植期间血流感染（BSI）的发生率为24.3％。移植后100 d的OS率为83.8％，100 d内DFS率为80.2％，100 d内NRM为16.1％，移植后100 d复发的累积发生率为4.5％。5例移植后出现新生低滴度抗体的患者移植后血象重建良好，100 d内全部生存。移植后抗HLA抗体MFI持续≥8 000的9例患者（其中4例患者DSA的MFI低）移植后进行PAIA联合RTX 2～4次治疗，3例出现抗体MFI下降，血象植入良好，并存活；持续无效的6例患者在100 d内有3例因感染死亡，1例因原发性植入失败死亡，上述死亡患者均有低丰度的DSA。此外，移植后有4例患者DSA阳性，MFI低，合并非DSA的抗体MFI低，移植后未进行再次脱敏治疗，上述患者移植后植入良好，并存活。

## 讨论

异基因造血干细胞移植仍然是恶性血液病的重要治疗手段之一。随着单倍体移植和HLA错配移植数量的增加，高HLA抗体导致的问题受到关注，前期研究表明预存DSA可能导致haplo-HSCT植入失败，也可以导致脐带血移植植入延迟[8]–[9]。高HLA抗体即使是非DSA也可能对移植的预后产生一定影响[10]。因此，对于HLA不匹配的供受者，如果存在高滴度抗HLA抗体，移植前脱敏治疗非常有必要。

迄今为止，清除抗体的主流方法主要包括血浆置换、静脉输注丙种球蛋白、RTX，硼替佐米及血小板输注近些年也被提及作为移植前脱敏治疗。实体器官移植中，血浆置换联合RTX为主要脱敏方法，目前关于移植前脱敏治疗尚无统一定论，现阶段主要采用抑制抗体产生、抗体中和及清除循环抗体等方法达到协同清除的效果。既往PAIA作为抗HLA抗体治疗的手段主要用于肾移植及类风湿疾病[11]–[13]。与血浆置换不同，PAIA具有以下特点：①能特异性清除致病抗体，尤其是IgG；②不需要额外的新鲜冰冻血浆，不良反应少；③允许更高的血浆容量治疗从而有效减少免疫球蛋白（PAIA治疗1次可有效清除>85％的IgG）[11]。有报道揭示，对于难治性多发性硬化复发患者，PAIA治疗后B细胞总数下降，与激素治疗组相比，PAIA治疗组B细胞亚群减少与临床疗效改善间相关性显著（*P*<0.001）[14]。因此，我们猜测PAIA治疗不仅可以清除循环抗体，也可以通过减少B细胞进一步减少抗体生成。Geyer等[15]应用PAIA联合RTX治疗ABO血型不相合肾移植患者，34例患者治疗后抗体滴度有所下降，成功进行移植，PAIA治疗后无患者出现明显不良反应，仅1例患者在RTX治疗后出现出汗等不适。因此，PAIA联合RTX在肾移植清除抗体治疗中具有良好的安全性，但仍需要大样本多中心研究证实，目前尚无报道揭示PAIA影响RTX血流动力学。目前有两例在造血干细胞移植中使用PAIA脱敏治疗成功的病例报道，Braun等[16]采用蛋白A吸附柱吸附致病抗体，Wilk等[17]通过移植后PAIA脱敏治疗促进植入成功。

本研究结果显示，PAIA联合RTX使抗HLA抗体总体的MFI丰度和不同位点抗体种类数量显著下降，可以有效清除抗HLA抗体。我们也观察了移植后HLA抗体情况，虽然前期PAIA联合RTX脱敏治疗对于大部分高致敏患者效果显著，但移植后仍有9例患者MFI持续升高，与移植后短期内供者B细胞不能替代受体组织中产生抗体的B细胞和浆细胞有关，也可能与异体细胞输注后激活原有的B细胞相关，其机制有待进一步研究。所有患者在PAIA治疗过程中未出现严重的不良反应，本研究也显示免疫球蛋白可以出现下降，与治疗前相比，IgG下降最为明显。

日本学者Takanashi等[10],[18]对386例接受清髓性预处理后行初次单份脐带血输注异基因造血干细胞移植患者进行回顾性分析，以确定抗HLA抗体可能产生的影响。结果显示，抗体阴性组、DSA组和非DSA组脐血移植后60 d粒细胞植入的累积发生率分别是83％、32％和73％，差异有统计学意义。本研究患者接受PAIA联合RTX脱敏治疗后，移植后28 d粒细胞及血小板植入的累积发生率分别为94.4％和82.9％。前期的研究揭示，在未预防性治疗CMV情况下，haplo-HSCT后CMV感染发生率为40％～70％[19]。本研究显示，患者移植后100 d内CMV感染的累积发生率为50.9％。既往报道显示，在接受造血干细胞移植的患者中BSI的累积发生率为9.2％～29.5％[20]–[21]。本研究中，患者移植期间BSI的发生率为24.3％，上述结果均与既往的报道相似。本研究所有接受吸附治疗的患者移植后100 d的OS率为83.8％。5例患者移植后出现低滴度新生抗体，对移植的预后无明显影响，但9例移植后抗体持续升高且合并DSA的患者中有4例死亡，因此移植后存在DSA且合并高丰度非DSA抗体的患者预后较差，目前例数太少未做统计学分析，有待扩增样本量进一步研究。

综上，本研究结果显示，PAIA联合RTX能够高效清除抗体，对HLA Ⅰ类及HLA Ⅰ+Ⅱ类抗体的清除效果显著，所有患者治疗过程未出现严重并发症。因此，在血浆资源非常紧缺的情况下，PAIA可作为脱敏治疗的替代疗法。移植前对抗体进行处理后，要警惕移植后抗体，持续的抗体监测非常有必要。然而，本研究为单中心小样本量研究，且移植后随访时间较短，结论尚需前瞻性多中心随机对照研究证实。
